# BioStar: An Online Question & Answer Resource for the Bioinformatics Community

**DOI:** 10.1371/journal.pcbi.1002216

**Published:** 2011-10-27

**Authors:** Laurence D. Parnell, Pierre Lindenbaum, Khader Shameer, Giovanni Marco Dall'Olio, Daniel C. Swan, Lars Juhl Jensen, Simon J. Cockell, Brent S. Pedersen, Mary E. Mangan, Christopher A. Miller, Istvan Albert

**Affiliations:** 1 Nutrition and Genomics Laboratory, Jean Mayer-United States Department of Agriculture Human Nutrition Research Center on Aging at Tufts University, Boston, Massachusetts, United States of America; 2 Inserm U915, Institut du Thorax, CHU Hôtel Dieu, Nantes, France; 3 National Centre for Biological Sciences, Tata Institute of Fundamental Research, GKVK Campus, Bangalore, India, and Department of Internal Medicine, Division of Cardiovascular Diseases, Mayo Clinic, Rochester, Minnesota, United States of America; 4 IBE, Institute of Evolutionary Biology (UPF-CSIC), CEXS-UPF-PRBB, Barcelona, Catalonia, Spain; 5 Bioinformatics Support Unit, Newcastle University, Newcastle, United Kingdom; 6 Novo Nordisk Foundation Center for Protein Research, Faculty of Health Sciences, University of Copenhagen, Copenhagen, Denmark; 7 Center for Genes, Environment and Health, National Jewish Health, Denver, Colorado, United States of America; 8 OpenHelix LLC, Bellevue, Washington, United States of America; 9 The Genome Institute at Washington University, St. Louis, Missouri, United States of America; 10 Bioinformatics Consulting Center, Pennsylvania State University, University Park, Pennsylvania, United States of America; University of California San Diego, United States of America

Although the era of big data has produced many bioinformatics tools and databases, using them effectively often requires specialized knowledge. Many groups lack bioinformatics expertise, and frequently find that software documentation is inadequate while local colleagues may be overburdened or unfamiliar with specific applications. Too often, such problems create data analysis bottlenecks that hinder the progress of biological research. In order to help address this deficiency, we present BioStar, a forum based on the Stack Exchange platform where experts and those seeking solutions to problems of computational biology exchange ideas. The main strengths of BioStar are its large and active group of knowledgeable users, rapid response times, clear organization of questions and responses that limit discussion to the topic at hand, and ranking of questions and answers that help identify their usefulness. These rankings, based on community votes, also contribute to a reputation score for each user, which serves to keep expert contributors engaged. The BioStar community has helped to answer over 2,300 questions from over 1,400 users (as of June 10, 2011), and has played a critical role in enabling and expediting many research projects. BioStar can be accessed at http://www.biostars.org/.

## Increased Adoption of High-Throughput Data and Barriers to Data Analysis

Recent years have seen rapid advances in high-throughput technologies in biology as evidenced by the types of experiments conducted and the number of different research groups now employing these technologies. This research typically uses data sets generated by high-throughput methods such as massively parallel sequencing, genotyping, gene expression analysis, and proteomics. These studies have the potential to influence research on human health through efforts like the 1000 Genomes Project, HapMap, the International Cancer Genome Consortium, and ENCODE. In addition, they are effecting change in other fields of study through initiatives like modENCODE, the Human Microbiome Project, the Human Plasma Proteome Project, and genome projects initiated by crop and livestock scientists.

Interpreting the results of such experiments requires a sophistication and infrastructure that traditionally have been available only to larger or well-funded centers, but investment in compute clusters and cloud computing have made computational resources increasingly accessible. The more difficult challenge currently faced by many research groups is a lack of readily accessible bioinformatics expertise. Addressing this flood of data requires a skill set largely absent from curricula at both the undergraduate and graduate levels, and most laboratories do not have the training and knowledge needed to perform their computational analyses unaided. Even experienced bioinformaticians often find themselves on unfamiliar ground as they strive to stay abreast of the rapid introduction of new technologies.

This problem is compounded by the growth of the bioinformatics field, which has produced thousands of tools, data resources, and web services over the last two decades. It is often laborious to identify tools with a specific functionality, as exemplified by more than 1,000 choices within the “Protein” category at Bioinformatics Links (http://www.bioinformatics.ca/links_directory/category/protein, accessed on 6 June 2011). Even when a tool can be identified, documentation and support are frequently missing or obsolete, and often have not anticipated more creative, advanced queries or novel implementations. As an example, the advent of workflow systems like Galaxy and Taverna [1], [2] enables users to weave customized pipelines together that employ multiple and disparate tools and data sources. Because questions and problems relating to workflow systems may cross traditional boundaries and domains of data and software providers, it can be difficult to find guidance from others with relevant experience. Though Taverna's “MyExperiment” and Galaxy's “Shared Pages” attempt to address this issue, questions about locating appropriate data, tools, or components beyond the current implementation remain best posed elsewhere. This lack of support can and often does lead to cumbersome bottlenecks during data analysis. Thus, it is essential to identify better ways of disseminating useful information—bits of code, expertise, advice—to individual researchers and to the community as a whole. It was with this goal in mind that BioStar was created.

## BioStar—A Bioinformatics Question and Answer Forum

BioStar promotes an active bioinformatics community by allowing researchers to pose questions and offer solutions to bioinformatics-related problems. BioStar was created in late 2009 by members of the Bioinformatics Consulting Center at Pennsylvania State University and is based on the Stack Exchange technology [3], which allows users to ask or answer questions for a given problem and aims to form a concise discussion limited to a single question. Questions and answers are rated by members of the site and can be edited by any member, in a manner analogous to a wiki. This platform has been used with marked success by programming and informatics communities on the popular web site http://stackoverflow.com/ [3]. Since its public launch on 18 January 2010, BioStar has grown quickly to a user base of 1,400 active registered participants as of June 2011 and has accumulated a knowledge base of over 2,300 queries related to bioinformatics and programming (Box 1). Each question has a mean of approximately three responses, while only a very small percentage (<0.5%) have no community-provided answer. Google Analytics software reports that at the time of writing, the site has served over 1.2 million page views and over 400,000 visits requested by 167,000 visitors.

**Box 1. Sample BioStar topics**

- Algorithm and database design and implementation
- Help choosing the appropriate software for a task
- Locating and utilizing biological databases
- Interpreting results, including choosing appropriate statistics
- Pointers to relevant publications and references
- Career advice

**Figure 1:**
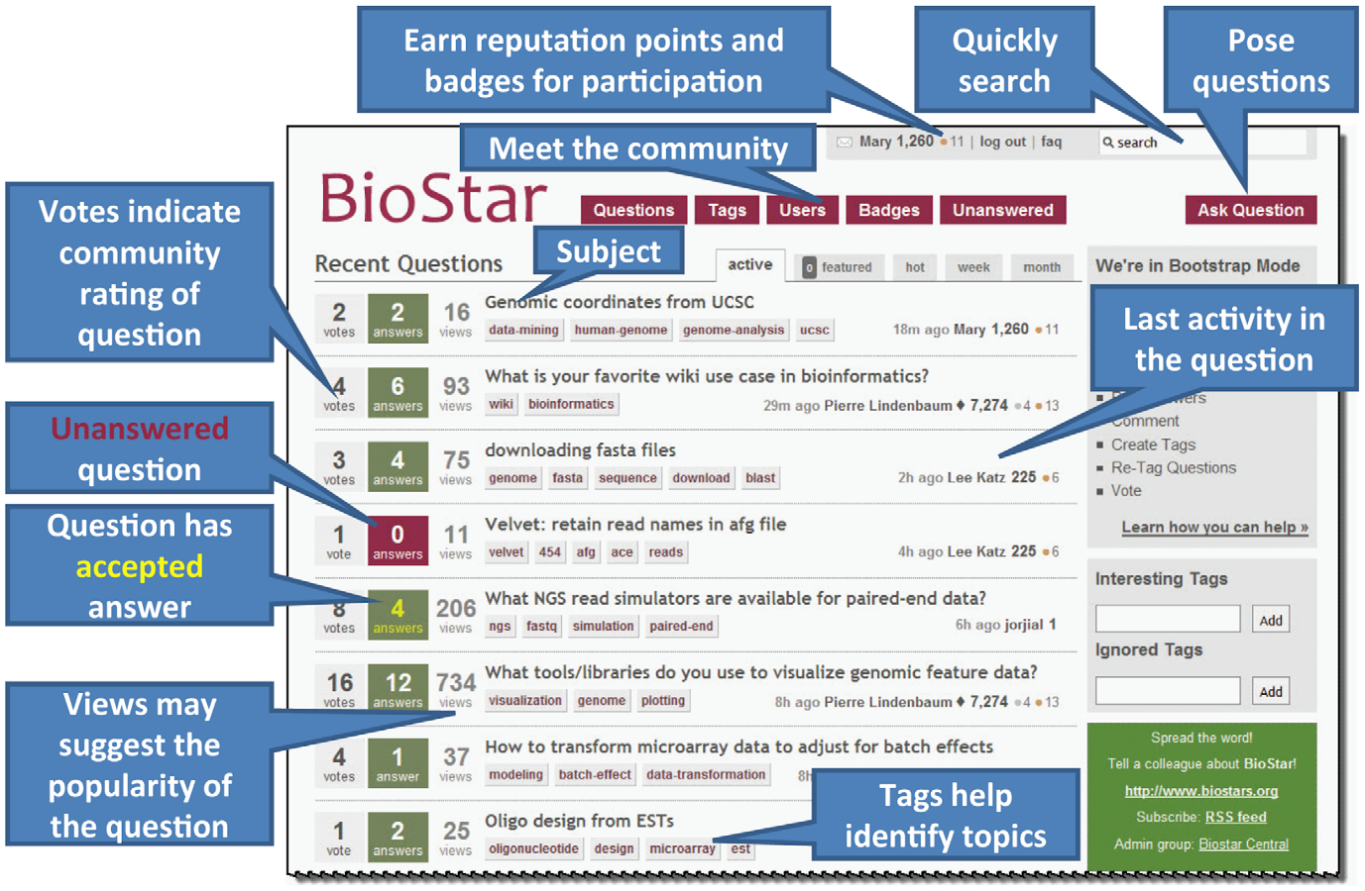
BioStar offers the opportunity to ask and answer a wide range of bioinformatics-related questions. Community members pose and answer queries on many topics. Questions and answers can be voted upon by members such that reputation points and badges are earned and help to assess the level of community respect and engagement. “Views” values can help assess the popularity of a question. Searches and tags enable users to quickly locate topics of interest.

The BioStar web interface provides an environment where inexperienced users are able to pose questions while engaging advanced users who are likely to assist in addressing those queries. The procedure required to propose a new technical question to the community is fast and simple, and poorly worded questions often see suggested rephrasing from more expert users. BioStar employs a system of user scores, badges, and privileges in order to encourage users to provide correct, pertinent, and useful answers. Thus, writers of both the initial question and those who provide a response are acknowledged for their efforts (Figure 1). Additionally, keyword tags coupled with a search tool make finding related questions or exploring a particular topic very easy compared to a traditional forum.

Another important feature of BioStar is the rewarding of good questions, correct or acceptable answers, and interesting comments with reputation points based on votes by other users. Although it is quite possible to ignore this notation, we have seen people increase participation to enhance their reputation, with the competition improving response rate and quality across the site. Based on votes, the most highly regarded answers for each question then percolate to the top. Although other sites based on the same technology were open to biologists, BioStar has benefited from the fact that bioinformaticians are probably more accustomed to this discussion format. In addition, early users have promoted the site through other online networks.

**Figure 2:**
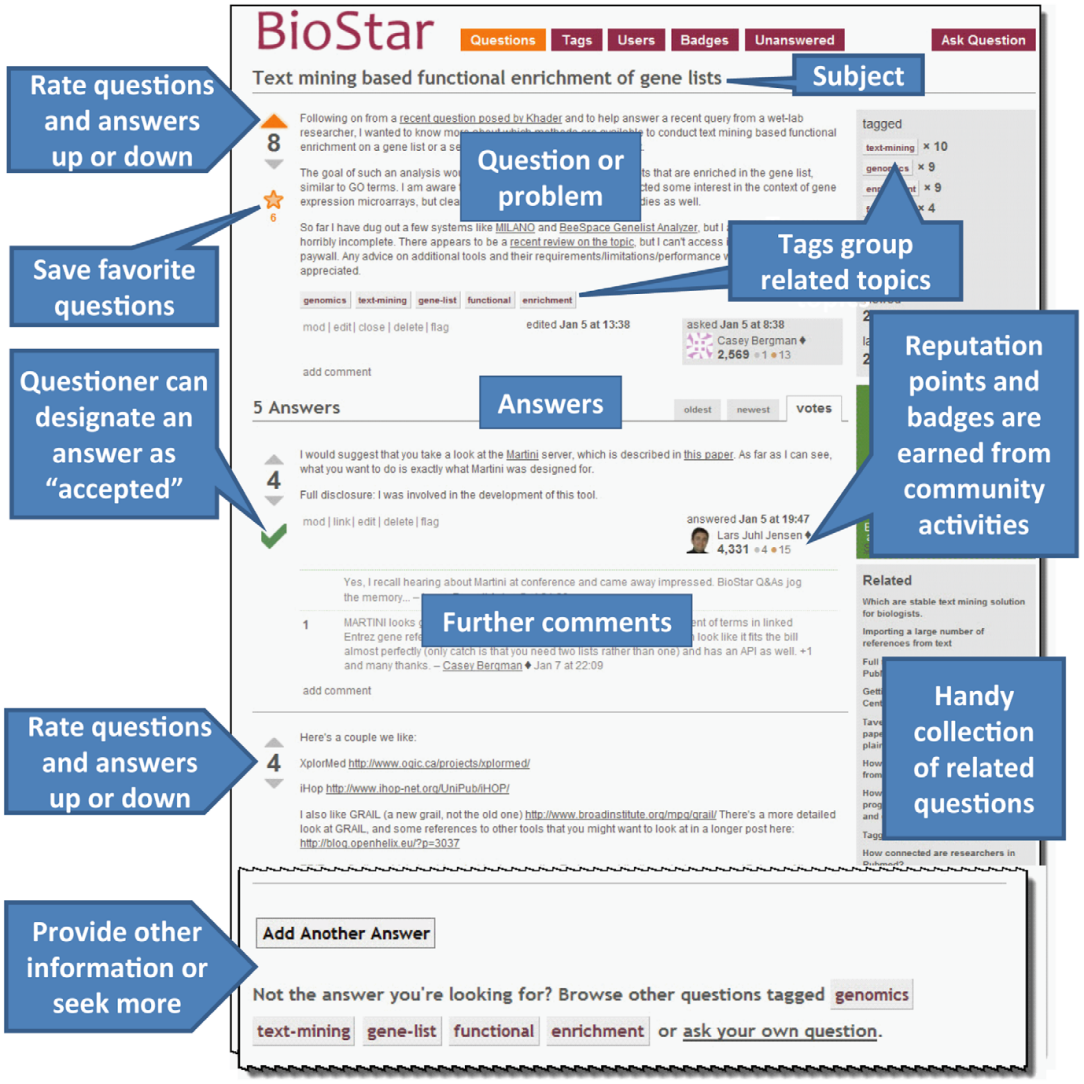
A typical discussion at BioStar. Discussion begins with a query, generates various answers, and yields additional comments that can lead to further information. Community voting on questions and answers contributes to reputation points and badges earned by users, along with activities such as editing and other types of participation. Question tags and automatically generated “related” questions can lead to further exploration of a topic. BioStar allows for additional answers to be appended at any time. Some questions acquire community “wiki” status if the issue is better served by a group discussion than by a discrete answer.

In addition to keyword tags coupled to a search tool and a rewards system based on votes cast by registrants, BioStar has other important advantages over traditional web forums. First, the community of BioStar users is both extremely active and growing at an astonishing rate. As a result, many questions receive an initial response within minutes of posting. Second, the Stack Exchange engine aims to make the threaded discussions brief and concise. In a conventional web forum, discussions are linear, consisting of a series of replies where the interrogating user can clarify his/her question in subsequent replies to the people who answer. However, this model tends to produce poorly readable archives of each discussion topic because it necessitates reading the entire thread in order to identify the exact question posed along with the correct or acknowledged answer. Stack Exchange sites make discussion easier to follow by limiting the discussion to a single question while a number of single-message answers are concatenated beneath that question, with highly regarded answers at the top (Figure 2). These force users to provide details needed to comprehend the question and make quality answers easy to find. Third, the user who asked the original question is encouraged to mark the answer he considers the most appropriate or correct. All these features contribute to easy perusal of the discussion archives by others interested in the same topic.

Accessible web-based forums, with content easily returned via search engines, are invaluable resources for students, early-stage career researchers, and experts looking for advice. The opportunity to learn from, and identify other researchers in the same field or with similar research questions can be invaluable for those in a specialized niche. Questions and answers may be technical and complex, or simply seek useful pointers, but often afford the significant advantage of feedback within a short amount of time. Questions are not queued in a help desk or forgotten within an e-mail inbox.

Much current biological research is complex and requires a multi-disciplinary approach, asking that a scientist be skilled in diverse disciplines, from programming to statistics and biology. Because it can take many years to become proficient in any of these fields, dialogue with colleagues is important in overcoming obstacles. For scientists who find it necessary to incorporate genomics or other computationally rendered data into their research objectives, a paucity of local expertise can cause a project to lose momentum. This is especially true in organizations that lack dedicated bioinformatics support staff. In such cases, a forum like BioStar offers a means for finding solutions that directly affect the way in which research proceeds. In other situations, questions posed on BioStar fall outside the scope of a bona fide collaboration and thus a dedicated bioinformatics core would not prioritize such an inquiry. In these instances, the collective expertise of BioStar users can provide informative and timely guidance that enable research to progress.

In summary, as a result of these combined properties, BioStar has become an active, dynamic, and fast growing online community for bioinformaticians and computational biologists. Having attracted a large and extensive set of users in a short time, it provides a point of reference for many smaller groups born around other social web sites and has had measureable impact on the manner in which certain research projects were undertaken.

## Use Cases and Success Stories

Topics recently added to the BioStar discussion represent a wide range of issues facing researchers. These include a discussion of “best practices” (http://biostars.org/search?q=bestpractices), determining which databases annotate data with specific data fields as needed for a particular analysis, advice on which programming libraries and tools are best suited for an experiment, feedback on a script or bit of code, help for poorly documented libraries, discussion of software and software pipelines (e.g., regarding genome scaffolding and exome sequencing; http://biostars.org/questions/6487/what-improvements-would-you-recommend-for-this-genome-scaffolding-software and http://biostars.org/questions/1269/what-is-the-best-pipeline-for-human-whole-exome-sequencing), and challenges to compare methods and tactics for solving a given problem. Furthermore, there are several documented cases where BioStar has contributed to completion of a research project or a peer-reviewed publication. BioStar has been used successfully to prepare a practical course on next-generation sequencing. Eleven different users submitted pertinent remarks and suggestions as to the content of the one-day, intensive course. These observations remain accessible on BioStar and the presentation that served as lecture material for the course itself is also available online (http://biostar.stackexchange.com/questions/3355, http://www.slideshare.net/lindenb/20101210-ngscourse). Advice from BioStar users on protein function and annotation assisted in writing an article about the role of *NOTCH2* in Hajdu–Cheney syndrome [4]. Questions on microRNA databases and tools aided a publication demonstrating the allele-specific effects of miR-522 on *PLIN4* and obesity [5]. In another case, the community helped researchers understand the specifics of proteomics data formats so that they could submit files to a journal as part of the review process [6]. These examples demonstrate the real impact that BioStar is having on its users and their research. In fact, one of the most popular questions on the site is about how to cite BioStar, and several publications in preparation are recognizing the site with a citation or acknowledgement.

## Outlook and Perspectives

The rate of biological data production has increased rapidly over the past decade, and this increase shows no signs of waning. Thus, while we expect the popularity of BioStar and other similarly modeled Q&A sites to continue, certain challenges need to be met in order to sustain interest and growth. The emergence of other Q&A sites, be they on niche topics or broadly based (e.g., SEQAnswers), argues that discussions among site managers on overlapping topics and cross-indexing of questions ought to take place. To this point, selected questions that were particularly relevant to specific user communities at other sites, particularly SEQAnswers and Stack Overflow, have been re-posted as an effective means to broaden the respective audiences. In any regard, all such Q&A sites must encourage that questions be pertinent to the theme(s) of that site and be clearly formulated in order that a response both wholly addresses the problem and provides for good search results. The community as a whole will need to attempt to redress issues while user etiquette will be a constant concern for site managers. Administration may become a real challenge as the site matures fully beyond the development and implementation phases and gains a large set of registrants.

We feel that the diligence and responsiveness of the BioStar community can be leveraged to solicit the expertise of still other experts, especially those in developing technologies and methods, and such will allow the site to flourish. One strategy in this vein thus far employed has been to contact resource provider teams to encourage their participation. Other outreach activities include a flyer developed by the community for users to post locally or at conferences and the use of social media to contact and encourage bioinformatics scientists to join BioStar.

BioStar does not aim to solve the shortage of bioinformatics expertise but to offer a forum for exchange of ideas, expertise, and knowledge in order to alleviate some of the shortcomings faced by researchers. We recognize that the platform may need to further evolve to meet the needs of the bioinformatics community. For example, frequently asked and related questions could be collated into guides or tutorials that would be listed under a separate section of the site. A separate category of posts may be established for announcements and requests for feedback on existing tools. A bioinformatics “blogroll” could be implemented to allow users to vote on various external bioinformatics blog posts or announcements, thus providing a community-based rating and filtering of the most current bioinformatics knowledge. In summary, Biostar enables bioinformatics knowledge-discovery and knowledge-sharing in an open, online ecosystem.
